# Impact of Local Intravitreal Injection Delivery on Cost, Carbon Emissions, and Attendance in Yellowknife, Northwest Territories

**DOI:** 10.1177/24741264261447502

**Published:** 2026-06-05

**Authors:** Annie Switzer, Gurkaran S. Sarohia, Eugene Michael, Mark Seamone

**Affiliations:** 1 MD Undergraduate Program, Faculty of Medicine, University of British Columbia, Vancouver, British Columbia, Canada; 2 Department of Ophthalmology and Visual Sciences, University of Alberta, Edmonton, Alberta, Canada

**Keywords:** fly-in, fly-out care model, ophthalmology, health economic outcomes, rural health services delivery, access

## Abstract

**Purpose:** To compare a remote retina clinic model to a referral-based model in terms of cost, compliance, and carbon impact for a fly-in, fly-out ophthalmology clinic in Yellowknife, Northwest Territories, Canada. **Methods:** Data were collected retrospectively from all living patients in the Northwest Territories who were referred for intravitreal injections from clinic inception in 2021 to November 2024. We estimated carbon emissions, costs, and appointment attendance for care delivered in Yellowknife vs care received via referral to Edmonton (Alberta Retina Consultants clinic). **Results:** A total of 171 patients were included. Carbon emission analysis (n = 171) showed a total mean difference of 12 6410 kg of carbon dioxide emissions used for patient travel between patients who received care locally (producing 77% lower emissions) vs those traveling to Edmonton. The estimated mean cost difference (n = 171) for patients receiving care in Yellowknife vs those traveling to Edmonton for care was more than CAD$1.6 million annually, with the majority attributed to transportation. Attendance analysis (n = 52) showed a higher rate of missed appointments in Edmonton vs Yellowknife (mean ± SD, 21.3 ± 14.4% vs 14.3 ± 15.6%, respectively). **Conclusions:** Delivering intravitreal injections locally in Yellowknife was associated with improved patient attendance to appointments, reduced costs, and reduced carbon emissions compared with retinal care provided via the traditional referral-based system in Edmonton.

## Introduction

The Northwest Territories encompass approximately 40% of Canada’s landmass but account for only 3% of the national population.^1^ The combination of vast geography with low population density poses substantial healthcare delivery challenges. In 2023, the Northwest Territories reported a deficit of CAD$265 million, due solely to medical travel expenditures.^2^ Furthermore, per capita spending in the territory in 2024 reached CAD$25,369, which is nearly 3 times the national per capita spending average of CAD$9,054.^3^ From 2022 to 2023 alone, more than 17 000 residents accessed nonemergency medical travel, generating more than CAD$30 million in associated costs.^4^

Fly-in, fly-out models have been implemented internationally to deliver specialty services to remote geographic locations. In Australia, an 11-member subspecialist ophthalmology fly-in, fly-out program was shown to reduce patient travel requirements and costs.^5^ Similarly, a junior ophthalmologist-led model delivered more than 12 000 intravitreal injections over 6.5 years across rural communities, thereby reducing the need for patients to travel.^6^ Comparable initiatives in Australia and Africa have demonstrated improvements in access to care, compliance, and health system efficiencies.^7–9^ Together, these programs provide a framework for adapting fly-in, fly-out ophthalmology services to remote populations in the Northwest Territories.

Beyond cost savings, fly-in, fly-out service delivery models have been associated with improvements in equity and chronic disease management by reducing geographic barriers to care. In the United States, mobile health units have been shown to save USD$30 to $36 per dollar invested by preventing emergency visits.^10^ Similarly, a general practitioner-led fly-in, fly-out clinic in Australia was associated with reductions in preventable hospital admissions, improved clinical outcomes, and decreased government expenditures.^11^ In the Northwest Territories, where access to specialty care frequently requires travel to Edmonton, fly-in, fly-out service delivery models may offer a framework to reduce care gaps while also mitigating some of the financial and logistical burdens of centralized referral-based care. These challenges are particularly relevant for retinal disease, in which treatment requires frequent and time-sensitive care.

Antivascular endothelial growth factor (anti-VEGF) injections are used to manage conditions such as diabetic retinopathy and macular degeneration and require administration every 4 to 8 weeks.^12,13^ Missed or delayed doses can result in disease progression and irreversible vision loss. Injection rates in Canada increased by 24% between 2019 and 2023, reflecting a rising demand for retinal services, yet access remains limited in remote and rural communities.^14^ Fly-in, fly-out retina clinics may improve treatment adherence, reduce missed appointments, and help prevent avoidable vision loss and downstream associated costs.

Since 2021, the fly-in, fly-out retina clinic in Yellowknife, Northwest Territories, Canada, has provided intravitreal injections and retinal assessments locally, eliminating the need for patients to routinely travel approximately 1500 km to Edmonton for care. In this study, we evaluated the cost, appointment attendance, and carbon impact of the remote clinic model and compared it with the traditional referral-based approach.

## Methods

### Setting and Design

We conducted a retrospective cohort study from the inception of the Yellowknife fly-in, fly-out retina clinic in 2021 until November 2024. At the clinic, all patients were treated using the treat-and-extend protocol in accordance with their interval between intravitreal injections, and each patient was seen by a retina specialist and received optical coherence tomography imaging at each visit. All living patients receiving intravitreal injections in the Northwest Territories were identified through the clinic-based electronic medical records system of Alberta Retina Consultants, the retinal service provider for Northern Alberta and the Northwest Territories. For geographic context, the referral center in Edmonton, Alberta, is approximately 1451 km from Yellowknife, and access for many residents requires air travel, which contributes substantially to the travel burden. Institutional review board/ethics approval (no. Pro00149313) was obtained for this study.

### Carbon Analysis

All living patients who received at least 1 intravitreal injection in Yellowknife, Northwest Territories, were included in the carbon analysis (n = 171). Travel distances were estimated using the shortest typical route each patient would take to reach Yellowknife or Edmonton. Land travel distances were calculated using Google Maps, while air travel distances were determined using the Air Miles Calculator.^15^ Carbon emissions for land travel were estimated using a standard emission factor of 400 g of carbon dioxide per mile. For air travel, emissions were calculated using the Open CO_2_ Network emissions converter.^16^ Emissions were calculated based on patients having attended the same number of appointments per year that they attended on average since inception.

### Cost Analysis

All living patients who had received at least 1 intravitreal injection in Yellowknife were included in the cost analysis (n = 171). Cost data were obtained from the Northwest Territories Medical Travel Department, including transportation and accommodation costs per night for both Yellowknife and Edmonton. The average number of clinic visits per patient per year since the clinic’s inception was calculated per patient. Total costs were estimated by multiplying the average number of annual visits by the corresponding travel and accommodation costs for each location, modeling a counterfactual scenario in which patients receiving care locally in Yellowknife would otherwise have traveled to Edmonton for the same number of appointments. Costs related to provider remuneration, clinic operations, medication procurement, transport and storage, and consumable materials were not included due to a lack of site-specific cost data and because these costs were expected to be comparable across care models.

### Attendance Analysis

All living patients who had received at least 1 intravitreal injection in Edmonton prior to the implementation of the Yellowknife clinic and had received at least 1 intravitreal injection in Yellowknife thereafter were included in the attendance analysis (n = 52). The nonattendance rate was calculated separately for appointments in Edmonton and Yellowknife by dividing the number of missed appointments by the total number of scheduled appointments and multiplying the result by 100 to obtain a percentage. Given that the data were not perfectly normally distributed, Welch *t* test was performed, with statistical significance being set at *P* values less than .05. Attendance rates were calculated using all scheduled appointments during each care period, rather than individual injections.

### Management of Emergencies

Patients received verbal postinjection instructions outlining symptoms that would require urgent assessment, including increasing pain, sudden vision loss, photophobia, or new floaters. Patients experiencing concerning symptoms between scheduled clinic visits were advised to call the Alberta Retina Consultants after-hours phone line, which is attended by a retina fellow or a retina specialist. If required, prompted escalation to urgent transfer to Edmonton for definitive retina management could be done through established medical travel pathways.

## Results

### Baseline Demographic Characteristics

A total of 171 patients received at least 1 intravitreal injection in Yellowknife over the study period (Table 1). The mean ± SD age of all attendees was 68.2 ± 13.2 years. Of the patients, 86 (50.3%) were female and 85 (49.7%) were male. The most common diagnoses were neovascular age-related macular degeneration in 57 patients (33.3%), diabetic macular edema in 30 (17.5%), and proliferative diabetic retinopathy in 24 (14.0%). Other diagnoses included branch and central retinal vein occlusion, choroidal neovascularization, and pachychoroid neovasculopathy (Table 1). Most patients (n = 74; 43.3%) were from Yellowknife, with smaller proportions from Hay River (n = 19; 11.1%), Inuvik (n = 13; 7.6%), and various other communities across the Northwest Territories and Nunavut. The average number of patient visits annually was 6.32.

**Table 1.** Baseline Characteristics of the Patients Who Received At Least 1 Intravitreal Injection at the Yellowknife Retina Clinic, Northwest Territories, Canada, 2021 to 2024 (N = 171).

Characteristic	Value
Age, y, mean ± SD	68.18 ± 13.2
Sex, n (%)	
Male	85 (49.7)
Female	86 (50.3)
Diagnosis, n (%)	
nAMD	57 (33.3)
PDR	24 (14.0)
DME	30 (17.5)
BRVO	10 (5.8)
CRVO	16 (9.4)
CNV	9 (5.3)
PNV	12 (7.0)
PCV	2 (1.2)
Myopic CNV	5 (2.9)
PDR and DME	3 (1.8)
CRVO and DME	1 (0.6)
nAMD and PNV	1 (0.6)
CRVO and DR	1 (0.6)
City, n (%)	
Yellowknife	74 (43.3)
Hay River	19 (11.1)
Inuvik	13 (7.6)
Tsiigehtchic	3 (1.8)
Fort Simpson	4 (2.3)
Norman Wells	4 (2.3)
Deline	6 (3.5)
Fort Smith	10 (5.8)
Wekweeti	1 (0.58)
Fort Liard	2 (1.2)
Fort Mcpherson	6 (3.5)
Colville Lake	2 (1.2)
Behchoko	7 (4.1)
Fort Providence	1 (0.58)
Fort Resolution	3 (1.8)
Enterprise	1 (0.58)
Wha Ti	2 (1.2)
Gameti	1 (0.58)
Tulita	2 (1.2)
Tuktoyaktuk	1 (0.58)
Ulukhaktok	1 (0.58)
Fort Good Hope	2 (1.2)
Aklavik	3 (1.8)
Lutsel ke	2 (1.2)
Wrigley	1 (0.58)

Abbreviations: BRVO, branch retinal vein occlusion; CNV, choroidal neovascularization; CRVO, central retinal vein occlusion; DME, diabetic macular edema; DR, diabetic retinopathy; nAMD, neovascular age-related macular degeneration; PCV, polypoidal choroidal vasculopathy; PDR, proliferative diabetic retinopathy; PNV, pachychoroid neovasculopathy.

### Carbon Emissions

**Figure 1.**
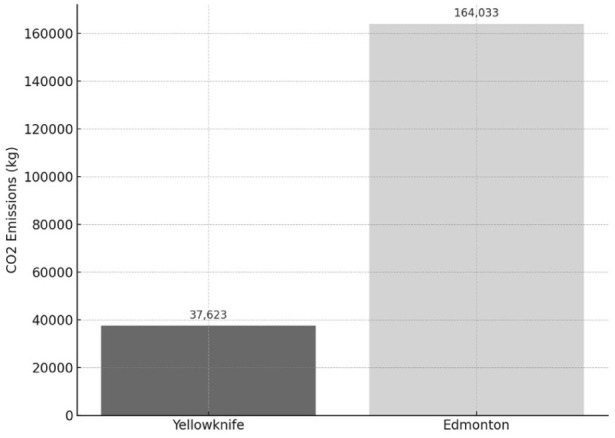
Annual carbon dioxide emissions (n = 171) related to patient travel to retina clinics for intravitreal injections in the Northwest Territories, Canada, 2021 to 2024, comparing local delivery in Yellowknife with referral to Edmonton. Dotted vertical line with values indicates the mean.

A total of 171 patients were included in the carbon emission analysis. The estimated annual carbon emissions for patient travel was a mean 37 623 kg of carbon dioxide emissions for those treated in Yellowknife and 164 033 kg of carbon dioxide emissions for those referred to Edmonton (Figure 1). This reflects a total annual mean difference of 126 410 kg of carbon dioxide emissions, with care in Yellowknife producing approximately 77% lower emissions than care requiring travel to Edmonton. Emissions were calculated based on average appointment frequency and standard emissions estimators for both air and land travel. This is equivalent to emissions produced by 29.5 gasoline-powered passenger vehicles driven for 1 year or the carbon offset from planting 2090 tree seedlings grown for 10 years.^17^

### Costs

A total of 171 patients were included in the cost analysis. The estimated total annual mean cost of return travel for all patients requiring intravitreal injection appointments was CAD$2,472,443 for those traveling to Edmonton, compared with CAD$849,677 for those attending the clinic in Yellowknife. The total mean cost difference between patients seen in Edmonton vs those seen in Yellowknife was CAD$1,622,766 annually.

**Figure 2.**
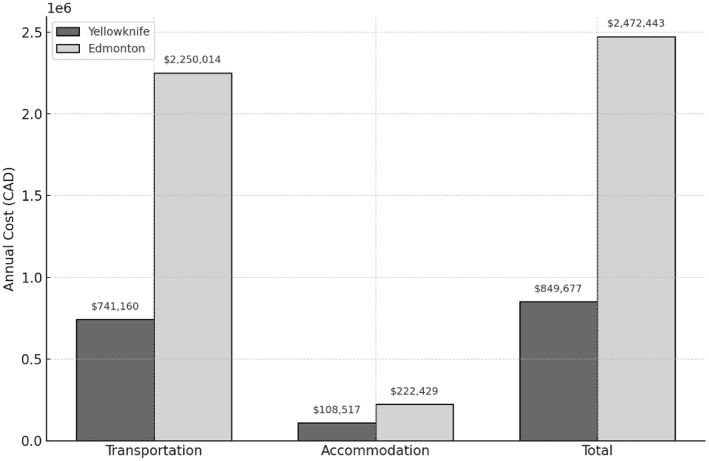
Annual transportation, accommodation, and total costs (n = 171) related to patient travel to retina clinics for intravitreal injections in the Northwest Territories, Canada, 2021 to 2024, comparing local delivery in Yellowknife with referral to Edmonton. Values are the mean cost, in Canadian dollars (CAD).

Much of this difference was attributable to transportation costs, which totalled a mean CAD$2,250,014 for Edmonton patients vs CAD$741,160 for Yellowknife patients. Accommodation costs contributed a smaller portion, at a mean CAD$222,429 for Edmonton patients and CAD$108,517 for Yellowknife patients (Figure 2). These values were derived from using standardized travel and accommodation rates from the Northwest Territories Medical Travel Department and the average number of annual visits per patient.

### Attendance

The attendance analysis included a total of 52 patients. These patients received at least 1 intravitreal injection in Edmonton prior to the implementation of the Yellowknife retina clinic and at least 1 intravitreal injection in Yellowknife thereafter. The nonattendance rate was found to be higher on average in Edmonton (mean ± SD, 21.3 ± 14.4%) than in Yellowknife (mean ± SD, 14.3 ± 15.6%). This difference in mean rates of missed appointments between the 2 care models was statistically significant (*P* = .020 by Welch *t* test) (Table 2).

**Table 2.** Comparison of Rates of Clinic Nonattendance by Clinic Location Among Patients Traveling for Intravitreal Injections in the Northwest Territories, Canada, 2021 to 2024 (N = 52).

	Nonattendance Rate		
Summary Statistic	Edmonton	Yellowknife	*P* Value, by Welch *t* test
Mean ± SD, % patients	21.3 ± 14.4	14.3 ± 15.6	.020
Median, % patients	19.0	10.0	

**Figure 3.**
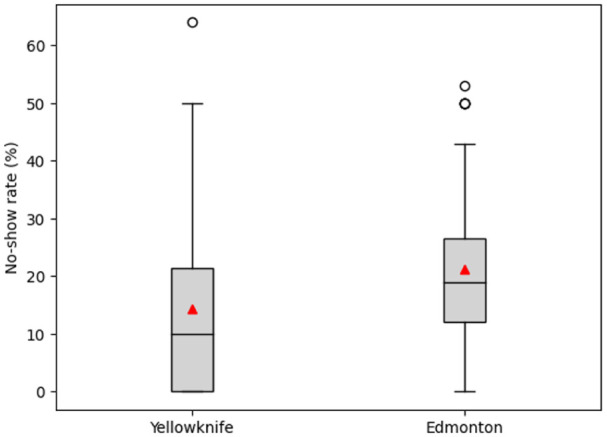
Distribution of median nonattendance rates (n = 52) by retina clinic location for patients traveling for intravitreal injections in the Northwest Territories, Canada, 2021 to 2024. Results are shown as box plots, where the horizontal line within the boxes indicates the median, boxes represent the interquartile range (25th–75th percentile), whiskers extend to values within 1.5 times the interquartile range, open circles denote outliers, and triangles within the boxes represent the mean.

Box plot analysis (Figure 3) further illustrated the difference in median nonattendance rates. Edmonton patients missed more appointments than Yellowknife patients, while Yellowknife patients showed a broader range and variability of nonattendance.

## Discussion

Our study compared the impact of delivering intravitreal injections locally in Yellowknife with the traditional referral-based approach of sending patients to Edmonton for intravitreal injections. We found that local delivery was associated with significantly lower travel-related costs, reduced carbon dioxide emissions, and improved patient attendance rates.

This reduction in nonattendance rates observed in Yellowknife is likely attributable to several factors, such as a reduced travel burden, improved accessibility, and stronger cultural and relational continuity of care. These factors are particularly relevant in northern Canada, where access to specialty healthcare is affected by reduced access to professionals and longstanding inequities in care delivery for Indigenous communities.

Prior studies from rural regions of Newfoundland, Ontario, and Saskatchewan have identified transportation, financial strain, and lack of provider continuity as barriers to accessing specialist care.^18–21^ In northern Ontario, patients in fly-in communities specifically reported difficulty establishing trust with transient healthcare professionals.^18^ The current fly-in, fly-out model in Yellowknife mitigates this by consistent and longitudinal presence of the same provider each month, which might partially account for the higher attendance rates observed.

Furthermore, treatment delays can lead to irreversible vision loss, particularly in conditions such as neovascular age-related macular degeneration, where anti-VEGF therapies are well established in preserving and improving visual outcomes.^22^ The improved attendance rates observed in Yellowknife, therefore, may translate into better long-term outcomes and visual preservation. This is supported by international studies. In Australia, patients living more than 100 km from a treatment center were significantly more likely to discontinue therapy, with dropout rates of 50%, compared with a dropout rate of 28% for those living closer.^23^ Other studies have shown that more than 10% of patients’ cancelled appointments were due to travel-related costs and logistical barriers.^24^ In Israel, missed appointments for anti-VEGF injections were significantly more common among patients living outside the hospital’s city.^25^

In addition to improved attendance rates, having retina care provided locally in Yellowknife resulted in savings of CAD$1.6 million in travel costs. Most of this difference was due to transportation costs, which are particularly high in northern Canada due to limited road infrastructure, long distances, and reliance on air travel. These findings align with previously published studies that show that care provided in clinics in rural and remote Canada is more cost-effective than centralized care. For example, an outreach psychiatry clinic in rural Manitoba functioned at 21% of the cost of the standard urban outpatient visits, which resulted in significant savings for the health system and patients.^26^ Similarly, in Australia, an outreach head and neck clinic saved a median of AUD$285 per patient visit as well as a reduction of AUD$215 in government reimbursements.^7^

The environmental impact of patient travel also differed between the 2 care models. Annual mean carbon emissions generated by patients traveling to Edmonton were estimated at 164 033 kg of carbon dioxide, as compared with estimated mean emissions of just 37 623 kg for those treated in Yellowknife, which marks a 77% reduction. These results align with national priorities such as the Canadian Medical Association’s commitment to decarbonization and achieving net-zero healthcare emissions by 2050.^27^

The breakeven point for fly-in, fly-out programs is important to consider from a health system perspective. Breakeven point occurs when the costs of providing specialist care locally are offset by reductions in patient travel expenditures, emissions, and missed appointments. This threshold is highly context dependent and is influenced by factors such as care frequency, travel distance, and the extent of public funding available. These models are most sustainable in regions with geographically dispersed populations that require recurrent specialty care, such as a retina service in ophthalmology, where patients require regular intravitreal injections. Although the specific cost structure identified in the Northwest Territories may not be directly generalizable, the underlying principles are relevant to other remote and rural settings where centralized referral models create significant logistical and economic challenges.

While cost and emissions reductions are important system-level outcomes, the effectiveness of fly-in, fly-out programs also depends on adequate clinic frequency and capacity to meet clinical demand. The Yellowknife retina clinic was structured to provide ongoing intravitreal therapy for chronic retinal disease rather than episodic or limited care, as reflected by observed appointment attendance across repeated visits. fly-in, fly-out models with insufficient service capacity may achieve low costs at the expense of clinical effectiveness, underscoring the importance of aligning clinic volume with population need. In this setting, the fly-in, fly-out model decreases travel requirements, missed appointments, and indirect expenses, thereby enhancing access to essential specialty care for individuals in rural and remote areas.

### Limitations

This study has some limitations. First, its retrospective design limits our ability to infer causality, and the data relied on existing administrative records, which may be subject to inaccuracies or incomplete reporting. Second, while we compared compliance, cost, and emissions, we did not assess clinical outcomes such as visual acuity or disease progression. However, anecdotally, we have not observed a large disparity in outcomes. Third, the cost analysis was limited to indirect patient-related expenses, specifically, medical travel and accommodation. Direct healthcare delivery costs, including provider remuneration, clinic operations, medication procurement, transport and storage, and consumable materials were not considered. A formal sensitivity analysis was omitted. Additional indirect costs, such as lost income, caregiver burden, and quality of life impacts, were also excluded. Further studies could evaluate whether improved compliance translates to measurable differences in visual outcomes. Finally, indirect costs such as lost income or quality-of-life measures were not captured, and their inclusion could further strengthen the case for local delivery.

## Conclusions

This study showed that delivering intravitreal injections locally in Yellowknife is associated with improved patient compliance, cost savings, and reduced carbon emissions compared with the traditional referral-based system in Edmonton. These findings support the value of decentralized care models in improving equity, efficiency, and sustainability in remote locations. Future research could evaluate long-term outcomes of patients treated in local vs referral-based settings and explore patient perspectives.
